# DEMENTIA CAREGIVERS, BURDEN, AND EMERGENCIES

**DOI:** 10.1093/geroni/igad104.1372

**Published:** 2023-12-21

**Authors:** Nicole Crawford, Bruce Levin, Amber Gum, Dinorah Martinez Tyson, Anthony Masys, Makeba Huntington-Symons

**Affiliations:** The University of South Florida, Tampa, Florida, United States; University of South Florida, Tampa, Florida, United States; University of South Florida, Tampa, Florida, United States; The University of South Florida, Tampa, Florida, United States; University of South Florida, Tampa, Florida, United States; Alzheimers Association, Florida Gulf Coast Chapter, Clearwater, Florida, United States

## Abstract

Caregivers of people living with dementia face multiple barriers, including social isolation, mental health problems, limited time for themselves, and limited support services. Navigating emergencies, including hospitalization, financial loss, and natural disasters, can exacerbate the burden that dementia caregivers face. This study examined dementia caregivers’ perspectives on burden, emergencies, and emergency preparedness. Semi-structured interviews were conducted with a predominantly female sample of fourteen dementia caregivers living in Florida. Data were analyzed using thematic analysis methods to define codes and identify themes. Results showed all dementia caregivers identified the term burden as having a negative connotation about their experience and the person living with dementia. Dementia caregivers predominantly stated that they cared for their family members out of love and a sense of responsibility. Findings also showed individual and caregiving experiences (e.g., being a single mom or having a previous caregiving role) prepared dementia caregivers to define and encounter emergencies. Understanding caregivers’ perspectives of burden, emergencies, and emergency preparedness is essential to developing evidence-based interventions and support services that aim to mitigate the adverse effects of emergencies on caregivers. Implications of using preferred terminology and recommendations for providing person-centered emergency planning will be discussed.

